# Lipid Metabolism and Obesity in Early Life: Drivers of Chronic Lung Disease From Development to Adulthood

**DOI:** 10.1002/mnfr.70427

**Published:** 2026-04-01

**Authors:** Jaco Selle, Natascha Mierswa, Leon Saschin, Jana Niehues, Jörg Dötsch, Eva Nüsken, Kai‐Dietrich Nüsken, Julia Schipke, Miguel A. Alejandre Alcazar

**Affiliations:** ^1^ Translational Experimental Pediatrics, Department of Pediatric and Adolescent Medicine Faculty of Medicine and University Hospital Cologne University of Cologne Cologne Germany; ^2^ Institute of Functional and Applied Anatomy Hannover Medical SchoolGerman Center for Lung Research (DZL) Hannover Germany; ^3^ Department of Pediatric and Adolescent Medicine Faculty of Medicine and University Hospital Cologne University of Cologne Cologne Germany; ^4^ Cologne Excellence Cluster On Cellular Stress Responses in Aging‐Associated Diseases (CECAD) and Center for Molecular Medicine Cologne (CMMC) Faculty of Medicine and University Hospital Cologne University of Cologne Cologne Germany; ^5^ Institute For Lung Health (ILH) Member of the German Center For Lung Research (DZL) University of Giessen and Marburg Lung Center (UGMLC) and Cardiopulmonary Institute (CPI) Giessen Germany

**Keywords:** chronic lung diseases, lung development and maturation, nutrition, obesity, perinatal programming

## Abstract

Overweight and obesity have become a severe global pandemic with growing social and health policy implications. Alarmingly, this not only affects adults, but also children and adolescents. Accumulating evidence in humans as well as in experimental studies indicates that an obesogenic environment across life span, ranging from in utero to adulthood, is associated with lung injury and chronic lung disease. Obesity is intimately linked to an excessive calorie intake as well as to an accumulation of lipids and a dysregulation of lipid metabolism in adipose tissue, but also within cells of many other organs. In the present review, we focus on the impact of lipids and lipid derivatives on lung morphogenic programming, normal and aberrant lung homeostasis as well as initiation and progression of chronic lung diseases. A specific emphasis was placed on alveolar surfactant and sphingolipids, and their functional contribution to molecular and cellular processes in the developing and mature lung. Finally, this review also discusses briefly the hitherto only little explored preventive and therapeutic possibilities of lipids and dietary adaptations, and thus the modulation of ingested lipids on lung health. Overweight and obesity, affecting all ages, contribute to lung injury and chronic lung disease. This review explores how lipids and their derivatives influence lung development, function, and disease progression—including surfactant and sphingolipids—and considers the underexplored potential of dietary lipid modulation as a preventive and therapeutic strategy for lung health.

## Graphical abstract

Metabolic and nutritional stressors are a growing global health concern. This review discusses how obesity, lipids, and lipid derivatives influence lung development, homeostasis, and chronic lung disease progression, highlighting roles of alveolar surfactant and sphingolipids. It also explores preventive and therapeutic strategies, including lipid modulation and dietary interventions, to support lung health throughout the lifespan in diverse populations and contexts.

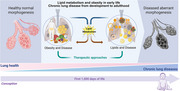

## 1 Introduction

This review focuses on the relationship between obesity and lipid metabolism and how these conditions contribute to normal lung morphogenesis, lung physiology, and the risk and progression of lung diseases. Therefore, we will discuss two key aspects: first, we will elaborate how obesity itself contributes to lung diseases, and second, we will explore how alterations in lipid metabolism, in part associated with an obesogenic environment, influence lung disease mechanisms and development. This review provides comprehensive insights into how excess body fat and disrupted lipid metabolic processes together shape disease risk and progression. This interplay is of particular interest as obesity is known to interfere with and modulate lipid metabolism [1, 2], thereby indirectly contributing to lung disease. The overarching topic of this review are the obsogenic conditions with a focus on lipids and nutrition across life span.

Overweight and obesity have become a severe global pandemic with growing social and health policy implications. In addition to polygenic causes, the combination of a sedentary lifestyle and high‐caloric diet favors an obese condition affecting an increasing rate of the population worldwide [3, 4]. Alarmingly, the shift toward an obesogenic lifestyle does not only affect adults, but also a growing number of children and adolescents. Therefore, obesity is an emerging risk factor for global health with a high attributable burden of disease in recent and future decades [3, 5, 6].

The World Health Organization (WHO) describes obesity as an abnormal or excessive accumulation of fat mass that ultimately causes a health risk. Although the body mass index (BMI) is not the most accurate measure of adipose tissue relative to body mass, it is widely used as an easily measurable indicator for obesity [7]. In adults, a BMI over 25 kg/m^2^ is considered overweight, while a BMI over 30 kg/m^2^ is classified as obese [7]. In children and adolescents, however, age‐ and sex‐specific BMI percentiles are used to determine obesity taking into account development and growth at this period of age [7, 8, 9].

The Global Burden of Disease (GBD) studies [5, 6, 10] analyzed the worldwide development of overweight and obesity in adults, adolescents, and children. These studies revealed that the prevalence of overweight and obesity has risen in all age groups, independent of ethnicity and socioeconomic background. Between 1990 and 2021, the prevalence increased by 155.1% and 104.9% in men and women, respectively, resulting in 2.11 billion individuals with overweight or obesity in 2021 [5, 10]. Based on these recent epidemiological data, the study predicts 3.8 billion adults will be affected by overweight and obesity in 2050. Alarmingly, this would account for more than half of the expected global population [5]. A similar trend is evident in children and adolescents, among whom the combined prevalence of overweight and obesity has doubled in the same time period as in adults [6], and a recent study predicts that by 2055, 186 million of 5‐ to 14‐year‐old individuals will be obese and 175 million of 15‐ to 24‐year‐olds [6].

The rising rate of overweight and obesity across ages is particularly concerning as it represents a risk factor for comorbidities. Although the impact of an obesogenic condition on metabolic and cardiovascular diseases has been studied extensively, the consequences for the lung and in particular the underlying molecular mechanisms are poorly understood. Initial studies indicate that overweight and obesity are not only risk factors, but may also function as modulators of progression and severity of various respiratory diseases such as asthma [11], chronic obstructive pulmonary disease (COPD) [12], idiopathic lung fibrosis (IPF) [13], and pulmonary hypertension (PH) [14]. Numerous studies in humans and rodent models have shown that overweight and obesity exacerbate systemic inflammation [11, 15, 16, 17], impair lung function, and increase airway stiffness and remodeling [11, 17, 18, 19, 20] resulting in a worse clinical outcome [11, 13]. However, the relationship is much more complex, as a phenomenon known as the “obesity paradox” can also be observed [21, 22]: Clinical studies with patients suffering from COPD or pneumonia show a positive correlation between BMI and lung function or survival [23, 24, 25], indicating that being overweight or obese may not only have adverse effects on chronic lung diseases. These observations highlight the complex interplay between excess of adipose tissue and respiratory pathophysiology [16, 26].

**FIGURE 1:**
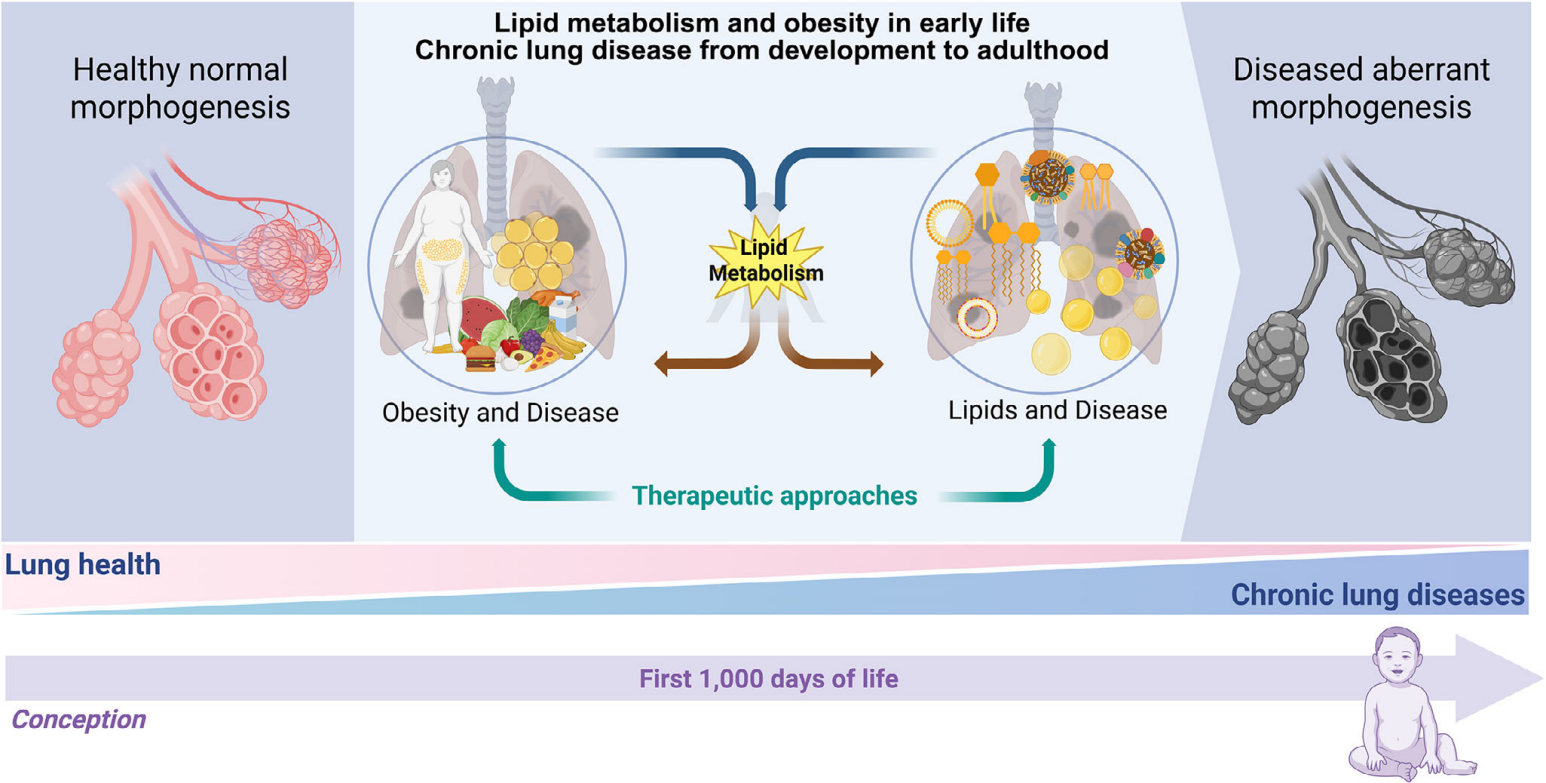
Scheme illustrating the overarching topic of this review with a focus (i) on how nutrition, lipid and derivates as well as obesity in early life disrupt lung homeostasis and contribute to chronic lung disease; and (ii) on possible preventive and therapeutic strategies. Created in BioRender. Alejandre Alcazar, M. (2026).

The following sections of this review focus on cellular and molecular processes in the lung under an obesogenic condition with a specific emphasis on lipids and nutrition across the lifespan (Figure 1).

## 2 Lung Health in an Obesogenic Environment

**FIGURE 2:**
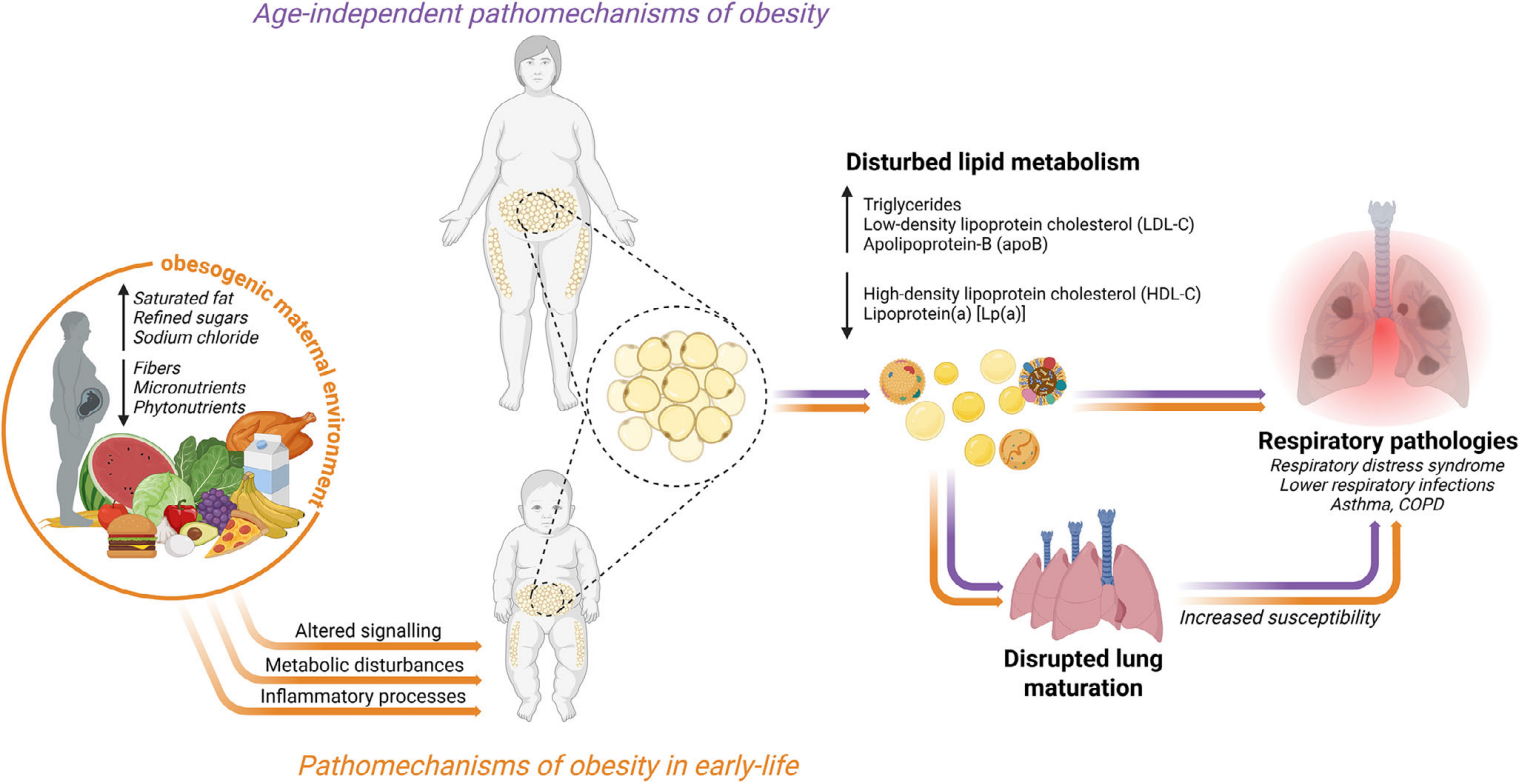
Schematic overview how maternal and early‐life obesity impacts lung maturation as well as susceptibility and pathogenesis of respiratory pathologies through changes in lipid metabolism. Created in BioRender. Alejandre Alcazar, M. (2026).

The following section discusses obesity‐associated pathomechanisms affecting the lung. First, we will briefly discuss obesity‐related mechanisms and then focus on the the implication of an obesogenic environment on lung health in early life (Figure 2).

### 2.1 Obesity, a Growing Social‐Economic Burden and Its Implication on Lung Health

According to the WHO, over 20% of pregnant women in Europe were classified as obese in 2022, with the prevalence continuing to rise [27]. Although maternal undernutrition remains a concern in low‐ and middle‐income settings, the global rise in overweight and obesity among women of reproductive age has emerged as a pressing public health challenge [5, 28, 29]. Here, also the relationship between low income or the limited access to education and dietary choices should be emphasized [30, 31]. Individuals with limited financial resources often face a relative higher cost when trying to maintain a healthy diet, which can lead to eating habits that promote obesity [30, 31]. The consequences of obesity extend well beyond the immediate pregnancy‐related complications such as gestational diabetes, preeclampsia, miscarriage, or stillbirth. At the same time, additionally to inflammatory responses as well as metabolic and lipid alterations, early exposure to an obesogenic environment can also have detrimental effects on disease susceptibility and progression. This increase in obesity is not only a result of excess calories that threaten the health of mothers and children, but is also associated with the declining quality of the modern diet. The Western‐style diet (WSD) is characterized by a high intake of ultraprocessed foods that are rich in saturated fat, refined sugars, and sodium chloride, combined with low intake of fiber, micronutrients, and phytonutrients. As the name suggests, this diet is particularly prevalent in industrialized nations and emerging economies undergoing rapid nutritional transitions [32]. The WSD not only promotes excessive gestational weight gain, but also fosters chronic low‐grade inflammation, metabolic dysfunction, altered lipid profile, and consequently adverse developmental programming, especially when exposure occurs during critical periods such as in utero or early childhood [29].

The pathomechanisms underlying obesity‐related respiratory complications are multifactorial. White adipose tissue is an autonomous endocrine organ secreting a variety of signaling molecules (e.g., adipokines), which have a significant impact on lung health [33]. Dysregulation of white adipose tissue homeostasis in individuals with obesity leads to white adipose tissue remodeling. Both, human and mouse studies provide evidence that pathogenic white adipose tissue is characterized by hypertrophic adipocytes and infiltration of inflammatory macrophages [34, 35]. As a result, obese white adipose tissue reacts with elevated secretion of pro‐inflammatory adipokines, e.g., TNF‐α, IL‐6, and leptin, whereas levels of anti‐inflammatory adipokines, such as adiponectin, are often reduced [33, 34, 35, 36]. This altered adipokine profile causes a systemic chronic low‐grade inflammation, which can act as a trigger or modifier of numerous comorbidities, including lung pathologies [37, 38]. The shift of adipokines favoring inflammation and metabolic disorders affects airway responsiveness, lung tissue remodeling, and immune response. These pulmonary effects related to obesity were shown in mouse models [39, 40, 41] as well as in humans [42, 43] and were linked to an increased susceptibility to inflammation including respiratory infections in individuals with obesity [44, 45]. An obesogenic condition can also perturb the gut or lung microbiome, causing disturbed immune‐microbiome interaction with adverse effects on lung health as shown in patients with asthma [46, 47]; however, the microbiome will be discussed in a separate chapter later on. Furthermore, obesity with dysregulation of lipid metabolism can alter the metabolome, contributing to destabilization of the lung structure [48, 49, 50]. For example, murine studies show that obesity‐related distinct alterations in the lung metabolome could also result in aberrant immune and cellular responses. Likewise, elevated glycolysis was identified as a typical obesity‐related metabolic adaptation. In line with these findings, the inhibition of glycolysis affected homeostasis of human airway smooth muscle cells and could have thereby an impact on airway hyperresponsiveness in asthmatic individuals with obesity [51, 52]. These metabolic and inflammatory processes possibly increase oxidative stress reaction in the lungs, a process that is relevant for lung homeostasis at any age [39, 45, 53].

The described processes associated with obesity are central at all ages and contribute significantly to the pathomechanisms of obesity‐related comorbidities. However, accumulating evidence suggests that maternal obesity initiates a series of interrelated developmental disruptions in the offspring, which collectively increase the risk of chronic diseases throughout life [17, 20, 54, 55, 56, 57]. Despite the growing knowledge, the long‐term health consequences for children, in particular for the pulmonary system, remain insufficiently addressed in routine obstetric care and public health strategies. Hence, we now explore the perinatal obesogenic effects on the child's lung maturation and development.

### 2.2 Early Life Obesity, Implication for the Developing Lung

The first 1000 days of life, starting with conception, represent a critical window for physiological development and long‐term health programming. During this period, key biological processes such as organogenesis, neurodevelopment, immune maturation, and metabolic imprinting are particularly sensitive to environmental cues [27]. Among numerous modulators, nutrition plays a pivotal role, extending beyond genetic inheritance. Maternal diet during gestation not only shapes fetal growth trajectories but also modulates the risk of disease development later in life through mechanisms such as epigenetic programming, altered endocrine signaling, and priming of the immune system [28]. After birth, early‐life nutrition continues to shape child's development and organ maturation through reinforcing or counteracting prenatal effects, thereby contributing to the establishment of metabolic and inflammatory set points that can persist into adulthood [17, 20, 55, 56, 57, 58, 59, 60]. It is well recognized that children of mothers with overweight or obesity have an increased risk of developing obesity, metabolic disorders and chronic diseases later in life [61, 62]. This concept, stating that extrinsic and intrinsic factors during pregnancy and early childhood influence the long‐term health of the child, is referred to as *perinatal programming* [63, 64, 65]. Embryos and fetuses of overweight or obese mothers are exposed to an obesogenic environment, both intrauterine and early postnatally [61, 66]. This includes not only a disturbed maternal metabolism, but also a chronic low‐grade inflammatory environment and exposure to an altered lipid profile [29]. Although mechanistic insights in human populations are limited, epidemiological evidence provides associations between maternal obesity and pulmonary sequelae. A large‐scale Swedish cohort study encompassing more than 1.8 million liveborn infants between 1992 and 2010 demonstrated a significant, dose‐dependent relationship between rising maternal BMI and infant mortality [67]. Particularly, post‐neonatal mortality rates were markedly elevated among children born to severely obese mothers. Over 80% of deaths were attributed to congenital anomalies, sudden infant death syndrome (SIDS), infections, birth asphyxia, and other neonatal complications, with congenital anomalies and SIDS being strongly associated with maternal BMI [67]. Furthermore, there is an increased risk of respiratory pathologies such as respiratory distress syndrome (RDS) and recurrent respiratory infections [68, 69, 70]. Maternal overweight and obesity are associated with a 7% and 16% increased risk, respectively, for lower respiratory infections (LRI) in children [71]. Moreover, a high maternal BMI correlates with an earlier onset of LRI, a higher frequency of infection episodes in the first three years of life, and longer hospitalizations due to respiratory complications [71]. When interpreting the association between maternal BMI, prematurity, and neonatal respiratory outcomes, it is important to consider that a high maternal BMI is closely linked to an increased risk of gestational diabetes mellitus (GDM) [72]. Studies demonstrate that maternal GDM elevates the risk of preterm birth in both nulliparous and multiparous women and is also a significant risk factor for neonatal RDS [73, 74]. These findings highlight the need to disentangle the respective contributions of obesity‐associated chronic inflammation and impaired glucose metabolism ‐including insulin resistanceto abnormal lung development, postnatal maladaptation, and the risk of neonatal respiratory disorders. These risks are further exacerbated in children with underlying allergic predispositions, suggesting an interplay between metabolic dysregulation, immune modulation, and respiratory vulnerability [71].

Similarly, experimental studies provide evidence that maternal high‐fat diet and obesity disrupt the development and maturation of the fetal lung through metabolic disturbances, inflammatory processes, and altered signaling cascades, predisposing the offspring to chronic respiratory disease [20]. In mice, maternal overnutrition during pregnancy and lactation results in an increased susceptibility of the offspring to various chronic lung diseases such as obstructive lung diseases and PH [17, 20, 75]. Various mouse studies have also identified persistent structural and functional abnormalities [17, 68, 75]. Offspring exposed to a high‐fat diet intrauterine and postnatally showed alveolar simplification characterized by larger alveoli and reduced septation, leading to a less efficient gas exchange [68, 75]. An obesity‐related impairment of lung development is dysanapsis, characterizd by the incongruent growth of airways compared to distal lung [76]. This structural concept has received increasing attention for its possible role in the developmental origins of obstructive lung diseases [76, 77]. Moreover, lung development in a perinatal obesogenic environment is accompanied by impaired angiogenesis and hyperproliferation of bronchial and vascular smooth muscle cells (SMC) [17, 20, 75]. This aberrant structural lung maturation is ultimately linked to reduced lung compliance, increased airway resistance, and reduced inspiratory capacity [17, 75].

The underlying pathogenic processes are affected not only directly by nutrition, but also indirectly by metabolic disorders and inflammation. For instance, metabolic disorders such as hyperinsulinemia and hyperleptinemia in pregnant mothers can interfere with insulin/leptin signaling pathways in the fetal lung: Under physiological conditions, leptin plays an important role by promoting the production of the surfactant protein A (SP‐A), which is important for lung maturation in the fetus [78, 79]. However, an elevated intrauterine leptin concentration regulates leptin receptors and impairs leptin signaling, which in turn compromises lung maturation and respiration after birth [75]. Moreover, a study in mice showed that high pre‐ and postnatal insulin levels promote insulin resistance in the offspring with subsequent inhibition of the PI3K/AKT/mTor signaling, a pathway central in cell growth, energy supply, and lung growth [80]. In rats, this disruption of the mTor signaling pathway led to the inhibition of Sftpa expression [81]. In addition, mouse studies underpin that a disrupted mTor signaling pathway reduces the proangiogenic VEGF (vascular endothelial growth factor) levels [82]. Suppression of these central developmental pathways affects normal morphogenic maturation of the lung with structural sequelae beyond infancy and childhood, possibly resulting in higher susceptibility to respiratory diseases later in life. Despite this growing evidence of obesity‐related detrimental effects on lung maturation associated with increased risk of lung disease in later life; the underlying cellular and molecular mechanisms are poorly understood.

However, obesity is not only characterized by an excessive fat accumulation, but is also associated with a profoundly dysregulated lipid metabolism. In an adipogenic state, patterns of both the quantity and composition of circulating and tissue‐bound lipids are changed. Major contributors to these patterns are cholesterol, low‐density lipoprotein (LDL) cholesterol, triglycerides, and high‐density lipoprotein (HDL). An imbalance in any of these factors can lead to dyslipidaemia, which is a condition of elevated triglycerides, increased low‐density lipoprotein cholesterol (LDL‐C) and elevated apoB, whereas high‐density lipoprotein cholesterol (HDL‐C) and lipoprotein(a) [Lp(a)] are reduced [37, 83, 84]. Against this background, the next section will focus on changes in lipids and lipid metabolism, emphasizing how altered lipid profiles contribute to the development and progression of obesity‐related diseases.

## 3 Lipids in Nutrition: Categories, Functions and Sources

Lipids is a collective term for water‐insoluble (hydrophobic) natural substances. Their insolubility in water is mainly due to the long hydrocarbon residues that most lipids possess. Unlike genes and proteins, which primarily consist of 4 nucleic acids and 20 amino acids, respectively, lipid structures exhibit a higher degree of complexity and comprise an extremely heterogeneous array of molecules in terms of structure and function. Lipids are involved in numerous cellular processes and thus play an essential role for the body homeostasis [85]. According to the LIPID MAPS classification system, lipids are divided into eight different categories [86, 87, 88] (see Table 1): (i) Fatty acyls, (ii) Glycerolipids, (iii) Glycerophospholipids, (iv) Sterol lipids, (v) Prenol lipids, (vi) Sphingolipids, (vii) Saccharolipids, and (viii) Polyketides.

**TABLE 1:** Lipid categories.

Lipid category	Structure	Example
Fatty acyls	Hydrocarbon chain with carboxyl group	Linoleic acid [89], fatty acids
Glycerolipids	Glycerol linked to one to three fatty acids	Triacylglycerols, TAGs
Glycerophospholipids	Glycerol linked to a phosphate group and (typically) two fatty acids	Dipalmitoylphosphatidylcholine Pulmonary Surfactant [90], Major component of biological membranes
Sterol lipids	Steroid nucleus (four linked hydrocarbon rings) with multiple variations	Cholesterol [91]
Prenol lipids	Five‐carbon isoprene units in different forms (e.g. linear, or cyclic) with multiple variations	Ubiquinones [92, 93]
Sphingolipids	Amino‐alcohol shingoid base (e.g., sphingosine) linked to different head groups	Ceramides [94], sphingasine, sphingosine
Saccharolipids	Sugar backbone linked to fatty acids	Defining gram‐negative bacteria: Glucosamine‐based lipid A [95], lipopolysaccharide (LPS)
Polyketides	Very diverse, derived from acetyl‐ or malonyl‐CoA	Macrolides [96], antibiotics or immunosuppressants

The provision of lipids to the body occurs through the ingestion of food‐containing fat, cellular de novo lipogenesis from carbohydrates taking place particularly in the liver, or the mobilization of stored lipids from lipid reserves, mainly the white adipose tissue. Lipids fulfil a broad spectrum of functions including membrane formation, energy storage, as well as cell signaling and inflammation.

Lipid droplets (LDs) are intracellular organelles specialized for lipid storage [97]. They consist of a hydrophobic core formed by neutral lipids surrounded by a phospholipid monolayer and various LD‐binding proteins [98]. In higher eukaryotes, many cell types harbor LDs. Adipocytes in white adipose tissue and hepatocytes contain many LDs reflecting their active lipid synthesis and storage, but also septal lipofibroblasts and alveolar epithelial type 2 cells (AT2) exhibit LDs as energy reserve and storage site of surfactant lipid precursors [99, 100, 101]. LDs are dynamic cellular organelles and act as a central hub in lipid metabolism and trafficking by coordinating intra‐ and extracellular lipid fluxes. Besides their obvious roles for energy maintenance and membrane homeostasis, LD biogenesis is closely linked to cellular stress responses including disturbed endoplasmatic reticulum (ER) homeostasis, oxidative stress, and starvation [102]. The sequestration of toxic lipid species or excess bioactive lipids in LDs is also an important defense mechanism against obesity‐related lipotoxicity [97, 102]. Lipotoxicity refers to the ectopic accumulation of lipid species in non‐adipocyte cells, leading to mitochondrial dysfunction, increased production of reactive oxygen species and ER stress, ultimately resulting in cellular death by apoptosis and local inflammation. These lipotoxic processes are associated with obesity‐related comorbidities like insulin resistance and metabolic syndrome [103, 104].

In the human lung adipose tissue is present within airway walls, and its tissue area is positively correlated with BMI and airway wall thickness [105]. Thus, “airway adiposity” may contribute to airway narrowing in respiratory conditions such as asthma, and exacerbate symptoms in obese patients with chronic lung diseases. Within the pulmonary gas exchange region, lipofibroblasts are specialized for lipid storage. They were first described in 1978 as one distinct interstitial fibroblast type observed during rat lung development, located at the base of newly formed septa [106]. Together with myofibroblasts they play a critical role for alveologenesis and epithelial cell differentiation [107, 108]. Proposed functional roles of lipofibroblasts include the storage of Vitamin A [109] and the provision of lipid precursors to AT2 cells for surfactant synthesis [110]. Moreover, lipofibroblasts are potentially involved in the development and progression of lung diseases such as bronchopulmonary dysplasia (BPD), IPF, and COPD due to their fibroblast growth factor 10 signaling [111]. Animal experiments suggest that the transdifferentiation of lipofibroblasts into myofibroblasts plays an important role in lung fibrosis formation, and that the manipulation of this process, e.g. by metformin, may be used for antifibrotic therapy [112, 113]. However, while in mice lipofibroblasts are ubiquitously present within septa, they are only rarely present in the adult human lung [100, 114], potentially limiting the transfer of these findings to the human situation.

In the epithelial cell population, AT2 cells harbor two lipid‐containing organelles: lamellar bodies and lipid droplets. Thus, the discrimination between these requires either marker‐based imaging or electron microscopy. AT2 cells of mammalian species including humans contain lipid droplets, albeit in low amounts under physiologic conditions [115, 116]. Different pathologic or environmental changes such as ischemia [117] or carbohydrate‐rich diets [99] were shown to induce an increase in AT2 cell‐related lipid droplet amounts in animal models, possibly influencing surfactant synthesis and composition [99].

## 4 Role of Lipids in Health and Diseases of the Lung

### 4.1 Physiological Role of Lipids and Surfactant in Lung Development and Maintenance of Lung Homeostasis

#### 4.1.1 Surfactant Composition, Function, and Synthesis in AT2 Cells

In the lung, lipids fulfil a special function as a main component of pulmonary surfactant. Surfactant is essential for lung function as it reduces the surface tension of the aqueous layer lining the alveolar surface area, thereby reducing the workload of breathing and preventing alveolar collapse, especially during expiration. Moreover, it serves as an immunological defense system against inhaled pathogens [118]. Thus, the surfactant system is highly relevant for lung health and disease at birth as well as later in life.

**FIGURE 3:**
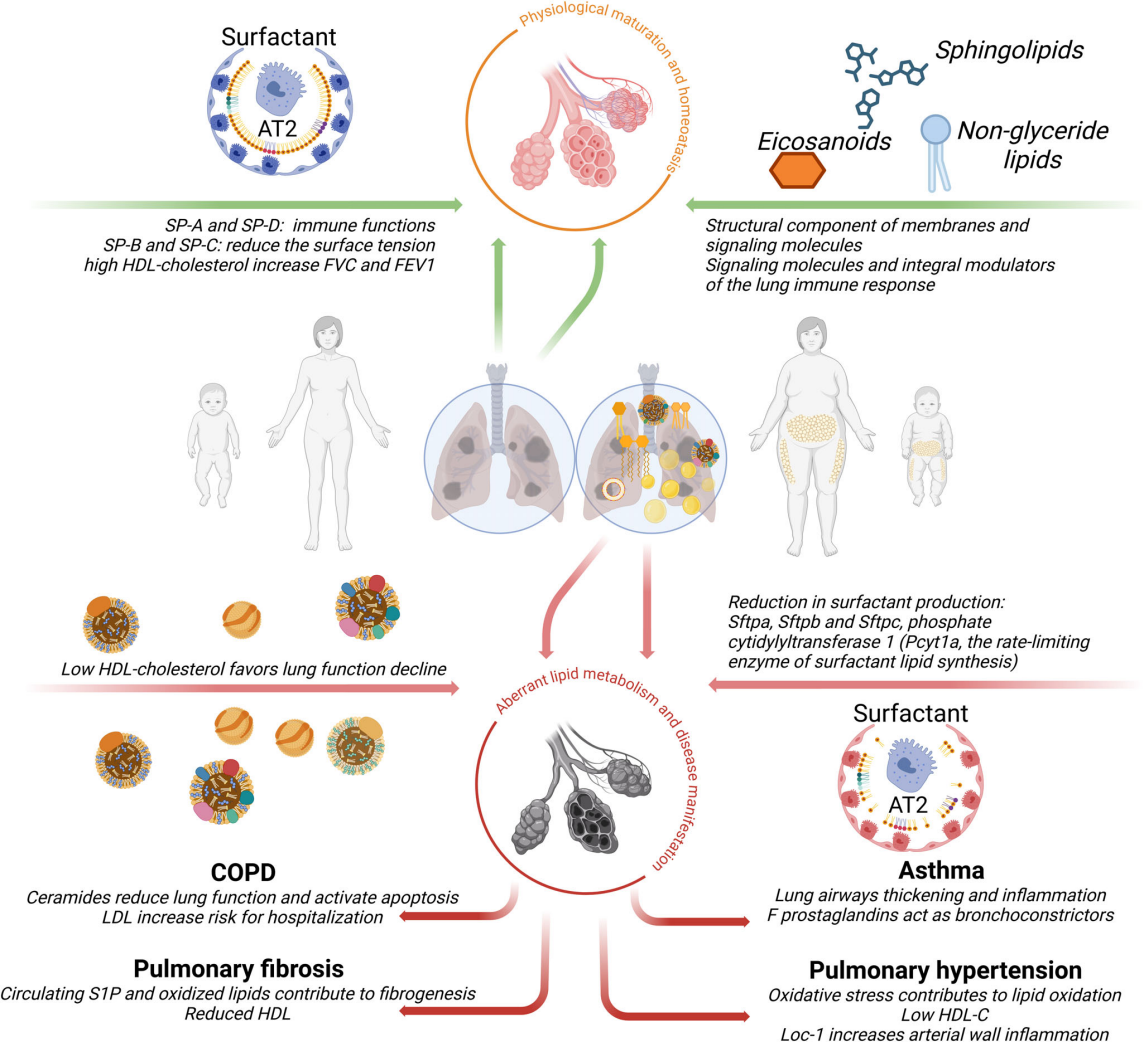
Overview of surfactant proteins and lipids in physiological lung maturation and homeostasis as well as lung pathologies. Created in BioRender. Alejandre Alcazar, M. (2026).

Pulmonary surfactant consists of ∼90% lipids by weight, mostly phospholipids. The main surfactant phospholipid is phosphatidylcholine (PC, ∼80%) with disaturated dipalmitoylphosphatidylcholine (DPPC, PC16:0/16:0) being the dominant constituent [90, 119]. Other phospholipids include acidic phosphatidylglycerol (PG), phosphatidylinositol (PI; 8%–15%) as well as small amounts of phosphatidylethanolamine (PE), phosphatidylserine (PS), lysophosphatidylcholine, and sphingomyelin [120]. Furthermore, surfactant contains 5%–10% neutral lipids, mainly cholesterol, and ∼10% surfactant proteins (SP‐A, SP‐B, SP‐C, and SP‐D). SP‐A and SP‐D fulfill immune functions, whereas SP‐B and SP‐C are essential for the reduction of surface tension [121, 122, 123]. Surfactant is synthesized, stored, and recycled in AT2 cells [118]. Approximately 45% of surfactant PC in adult mammals originates from de novo synthesis, but only limited knowledge exists about the provision of synthetic precursors for surfactant lipid synthesis [124]. From a biochemical perspective, the fatty acids (FA) moiety of phospholipids may be derived from either cellular de novo synthesis, uptake of free FA or lipoproteins from the circulation. In vitro experiments demonstrate that AT2 cells bind and internalize lipoproteins such as HDL, LDL, and very‐low‐density lipoprotein (VLDL), and utilize the contained FA for phospholipid synthesis [125, 126, 127]. The addition of lipoproteins increases intracellular PC synthesis and PC secretion in AT2 cells [126, 127]. Moreover, in a prospective human cohort study, high HDL‐C (HDL‐cholesterol) levels have been associated with improved forced vital capacity (FVC) and forced expiratory volume (FEV1), while low HDL‐C levels correlate with a faster decline in lung function [128]. In particular, surfactant cholesterol appears to be dependent on circulating lipoproteins. 83% of the cholesterol in rat lungs originates from lipoproteins previously taken up from the circulation [129]. In isolated perfused rat lungs, radiolabeled cholesterol is incorporated into lamellar bodies and released into the airspace together with PC [130]. The interplay between these various sources is incompletely understood and could be affected by levels of accessible substrates, nutritional factors, and distinct physiological conditions including overweight and obesity (Figure 3).

#### 4.1.2 Surfactant and Obesogenic Conditions

Accumulating evidence indicates that the surfactant system is affected in individuals with overweight or obesity, potentially contributing to a progressive obesity‐related lung function decline. An altered supply of circulating lipids could play a major role here. For example, experimental studies show that diet‐induced hyperlipidemia in rats leads to increased PG and decreased PE amounts in alveolar surfactant. The percentage of PG correlates with circulating FA concentrations, pointing to a direct influence on enzymes involved in phospholipid synthesis [131]. Similarly, the targeted supply of certain FA species leads to measurable shifts in the surfactant PC composition in rats. Specifically, exogenous C14:0 increases myristoylated PC species (PC16:0/14:0, PC14:0/14:0) at the expense of DPPC [132]. Similarly, an excessive fat intake results in increased amounts of DPPC in lung tissue and alveolar surfactant of newborn rats, which is associated with a reduced dynamic and static lung compliance [133]. Moreover, feeding adult mice a high‐fat diet alters the expression of enzymes involved in surfactant synthesis, and changes the surfactant phospholipid composition. This is accompanied by viscoelastic changes in lung mechanics and a reduced adsorption capacity of isolated surfactant at the air‐liquid interface [99].

The relationship between surfactant lipid composition and function is complex and incompletely understood. The molar proportions of rather rigid lipid species, such as DPPC, and rather fluidizing phospholipids, such as PC16:0/16:1 and PC16:0/14:0, are associated with the surface properties of surfactant [134]. Furthermore, surfactant lipids containing myristic acid have immunomodulatory functions [135, 136] and reduced amounts of myristoylated PC species may contribute to the increased susceptibility of obese individuals to SARS‐CoV‐2 infection [137]. Even minor changes in surfactant composition may become challenging during physical exertion or under conditions of limited respiratory capacity e.g., due to proinflammatory diseases such as allergies or asthma [138].

Only a few studies have examined surfactant changes in relation to body weight in humans. Brandsma et al. analyzed induced sputum, a secretion that largely corresponds to alveolar surfactant regarding its lipid composition [139] from a total of 41 adult subjects (29 men, 12 women) with a BMI ranging from 18.9 kg/m^2^ (normal weight) to 32.0 kg/m^2^ (grade I obesity) [140]. Here, a high BMI was correlated with increased absolute lipid concentrations and specific enrichments of diacyl‐glycerophosphocholines, ‐inositols, and ‐glycerols. In contrast, an analysis conducted by Tharp et al., of bronchoalveolar lavage fluid from 14 adult subjects (7 men, 7 women) with a BMI ranging between 24.3 kg/m^2^ (just within the normal weight range) and 50.9 kg/m^2^ (grade III obesity) revealed only minor changes in the alveolar lipidome with regard to the relative frequency of lipid classes or the fatty acid composition of lipid species [141]. Since the cohort studied by Tharp et al. predominantly consisted of individuals with overweight or obesity (13 of 14) the extent of obesity per se might not lead to significant changes in the pulmonary lipid metabolism. Conversely, Brandsma et al. by comparing a broader weight spectrum, suggest that body weight does affect pulmonary surfactant [140].

#### 4.1.3 Surfactant in Normal and Aberrant Lung Maturation

Lung development, and in particular the maturation of the surfactant system, begins during pregnancy. In women with obesity, a high maternal pre‐pregnancy BMI is associated with reduced levels of circulating phospholipids, sphingomyelin, and triacylglycerol species during pregnancy, suggesting an altered substrate supply for the initiation of surfactant synthesis in the fetal lung [142]. The maturation of the surfactant system in late pregnancy is influenced by changes in the intrauterine environment, including the availability of oxygen and nutrients [143, 144, 145]. In sheep, maternal overfeeding during late pregnancy results in a lower numerical density of AT2 cells and a reduced mRNA expression of *Sftpa*, *Sftpb*, and *Sftpc*, phosphate cytidylyltransferase 1 (*Pcyt1a*, the rate‐limiting enzyme of surfactant lipid synthesis), and glucose transporters (*Slc2a1*, *Slc24*) in the fetal lung, indicating a reduced ability to produce surfactant [70]. In the same line of evidence, intravenously injected radiolabeled VLDL crosses the placenta in pregnant rats and stimulates fetal PCYT1A activity and DPPC synthesis [146].

In addition to its impact on the developing surfactant system, maternal obesity is also a risk factor for preterm birth [147]. Since a functional surfactant system is of particular importance for the postnatal adaptation and the initiation of lung breathing at birth, the immature lung of preterm infants are at high risk for RDS, one of the main causes of prematurity‐associated morbidity and mortality [148]. An important aspect independent of prematurity that interferes with physiological lung maturation is nonfunctional surfactant caused by genetic alterations. Interestingly, linking prematurity and genetic alterations, SP‐C allele polymorphisms has been linked to very premature birth and RDS in a Finnish cohort of preterm and term infants [149]. In genetically modified mouse models, the deficiency in SP‐C results in progressive pulmonary disease associated with emphysema, a surplus of alpha‐smooth muscle actin indicating myofibroblast transdifferentiation, and monocytic infiltrates in the parenchyma, as well as epithelial cell dysplasia in the bronchial airway system [150]. Furthermore, mouse strains with a SP‐C deficient phenotype and the associated lung pathology resemble the conditions seen in patients with familial IPF caused by mutations in the *Sftpc* gene [151]. Furthermore, mice with an AT2 cell‐specific conditional deletion of ABCA3, which is responsible for lipid transfer into AT2 lamellar bodies, disrupts surfactant homeostasis in both newborn and adult mice; similarly, mutations in ABCA3 in humans are among the most prevalent group of mutations associated with surfactant‐related lung disorders [152, 153, 154, 155].

#### 4.1.4 Impact of Lipids beyond Surfactant on Lung Homeostasis

**Non‐glyceride lipids**, including (glyco)‐sphingolipid**s**, are involved in cellular processes such as differentiation, senescence, proliferation, and signaling [156, 157, 158]. Glycosphingolipids are structural components of membranes and signaling molecules that regulate cellular responses, and thus represent an integral modulator of the lung immune response. In humans, sphingosine kinase 1 (SphK1) promotes growth of glioblastoma by increasing inflammation mediated by the NF‐κB/IL‐6/STAT3 and JNK/PTX3 pathways [156, 159, 160, 161]. Especially for ceramides and its derivatives such as sphingosine‐1‐phosphate (S1P), a balanced level is necessary for lung homeostasis: ceramides are known to cause cell cycle arrest and apoptosis by ceramide‐induced caspase‐3 activation [158], while S1P facilitates proliferation by activating the Ras and ERK/MAPK signaling (concept of opposing roles, known as sphingolipid rheostat, see details in chapter 5) [156, 162, 163, 164]. In the lung, ceramides or glycosphingolipids contribute to the maintenance of the endothelial barrier [165].

**Eicosanoids**, a group of biologically active lipids, play a vital role in regulating inflammation and other physiological processes [166]. Prostaglandin E_2_ signals through four known G‐protein‐coupled receptors, EP_1‐4_. While EP_1_ and EP_3_ primarily act through calcium signaling, EP_2_ and EP_4_ increase cAMP levels in the target cells [167]. Thus, it has been demonstrated, that applying prostaglandins showed protective effects against lung fibrosis in a murine bleomycin‐induced lung fibrosis model [168, 169]. In asthma, prostaglandin is involved in allergic lung inflammation, and prostaglandin D_2_ (PGD_2_) and prostaglandin F_2_α (PGF_2_α) are mainly released by mast cells during allergic or inflammatory responses leading to bronchoconstriction [170, 171, 172].

### 4.2 Pathological Role of Lipids and Surfactant in Chronic Lung Diseases

Having described the important role of lipids in lung development, homeostasis, and maintenance of alveolar integrity, the next chapter will highlight the impact of lipids and lipid‐derived mediators such as ceramides, S1P or lipoproteins in lung‐related diseases [119].

Experimental mouse studies demonstrate that the disruption of the pulmonary lipid balance can trigger inflammation [173]. Moreover, lipid‐derived metabolites such as prostaglandins contribute to the onset of inflammation, but also play a crucial role in regulating its persistence and resolution through specialized proresolving lipid mediators (SPMs) such as maresins, lipoxins, resolvins, and protectins [174, 175]. For example, the maresins 1 receptor MaR1 negatively modulates the activity of the ion channel TRPA1 and thereby blocks inflammation [176]. However, imbalances in the levels of SPMs have been associated with various inflammatory conditions, including COPD [177]. Furthermore, in murine AT2 cells, the inactivation of sterol‐response element‐binding proteins (SREBP) via the interaction with insulin‐induced gene 1 and 2 (*Insig1* and *Insig2*) enhances lipogenesis, which can trigger pulmonary lipotoxicity [178].

In the context of **pulmonary fibrosis,** intratracheal instillation of oxidized PC species in mice induces a severe fibrotic response. This suggests that altered and oxidized lipids secreted by AT2 cells promote a profibrotic,M2‐like phenotype by reprogramming of local macrophages, ultimately contributing to fibrogenesis [179]. Circulating lipids such as S1P have been identified to be important in fibrosis development and progression in the lung and other organs [180, 181]. Repeated injury activates repair responses in AT2 cells, prompting them to secrete profibrotic cytokines and driving lung fibroblasts to proliferate and differentiate into myofibroblasts. These myofibroblasts are capable of producing extracellular matrix (ECM) [182, 183, 184], which ultimately can disrupt alveolar architecture [185], establishing AT2 cells and fibroblasts as key regulators of IPF progression. Furthermore, dysregulated lipid metabolism in IPF impairs AT2 cell repair and promotes the differentiation of fibroblasts into myofibroblasts [186, 187, 188]. In human observational studies, the concentration of HDL particles correlates inversely with IPF severity and prognosis [189]. Additionally, low HDL cholesterol levels have been associated with fibrosis progression and worse outcomes [190].

In **COPD**, the dysregulated level of lipid derivatives has been observed to be deeply intertwined with disease progression [177]. In human population‐based studies, specific ceramides and sphingomyelins were linked to reduced lung function, with certain ceramides being associated with lower FEV1 values and higher prevalence for COPD [191, 192, 193]. Ceramide inhibits efferocytosis, potentially contributing to lung destruction by triggering apoptosis via a caspase‐3 activation. Moreover, apoptotic cell clearance is impaired by ceramide‐induced attenuation of Rac1 plasma membrane recruitment of macrophages [194].

Several observational studies have indicated a link between the use of statins (drugs that lower LDL cholesterol) and a reduced risk of exacerbations and hospitalizations related to COPD [195, 196]. Moreover, a meta‐analysis of randomized controlled trials reported improvements in both exercise capacity and lung function among COPD patients under statin therapy [197]. However, the two largest double‐blind randomized controlled trials examining the impact of statins on the frequency and severity of COPD exacerbations have yielded conflicting outcomes [198, 199, 200]. Although one of these trials observed a reduction in the rate of COPD exacerbations [199], the overall findings remain inconsistent and needs further exploration in the future.

In **asthma,** lipid‐derived metabolites such as prostaglandins are central modifiers of disease progression [201, 202, 203]. They have been shown to play a dual role. E prostaglandins are potent bronchodilators, and EP_2_ and EP_4_ receptors lead to higher intracellular cAMP concentrations, resulting in smooth muscle relaxation. In contrast, F prostaglandins are bronchoconstrictors due to an increase in intracellular calcium levels [204, 205].

The white adipose tissue deposition within the airway wall is related to BMI, wall thickness, and inflammatory cell numbers, suggesting an obesity‐related effect on airway pathophysiology and immune response [105]. Notably, SP‐D, one of the surfactant proteins implicated in innate immune function, binds directly to the surface of eosinophils and inhibits the formation of extracellular traps, thereby reducing airway inflammation. Thus, SP‐D might serve as a potential therapeutic modifier in asthma [206].

**Pulmonary hypertension** is intimately linked to elevated oxidative stress that in turn contributes to lipid oxidation [207]. These oxidized lipids are involved in several key pathophysiological features of PH. In arteries of several animal species and humans, an upregulation of lectin‐like, oxidized low‐density lipoprotein receptor‐1 (LOX‐1) was observed. LOX‐1 colocalizes with apoptotic endothelial cells and induces an accumulation of oxidized lipids in arterial walls triggering inflammation [208, 209, 210].

Inflammation is a central mechanism in PH, but also in the pathogenesis of arteriosclerosis of pulmonary arteries. Arteriosclerosis is often driven by lipid uptake into macrophages and foam cell formation [211, 212]. In human idiopathic pulmonary arterial hypertension (PAH), a significant reduction in plasma HDL‐C was reported [213, 214], with lower levels of HDL‐C correlating with a higher mortality [215].

Collectively, there is a growing body of evidence that lipids, lipid mediators as well as lipid derivatives are modifiers in the progression and outcome of chronic lung diseases. Deeper insight in the complexity of lipid‐lung and lipid‐immune interplay may offer new preventive strategies for maintenance of lung health as well as novel therapeutic avenues for chronic lung diseases.

## 5 Lipids and Their Metabolism as a Therapeutic or Preventive Avenue

### 5.1 Therapeutic and Preventive Pharmacological Treatments

**FIGURE 4:**
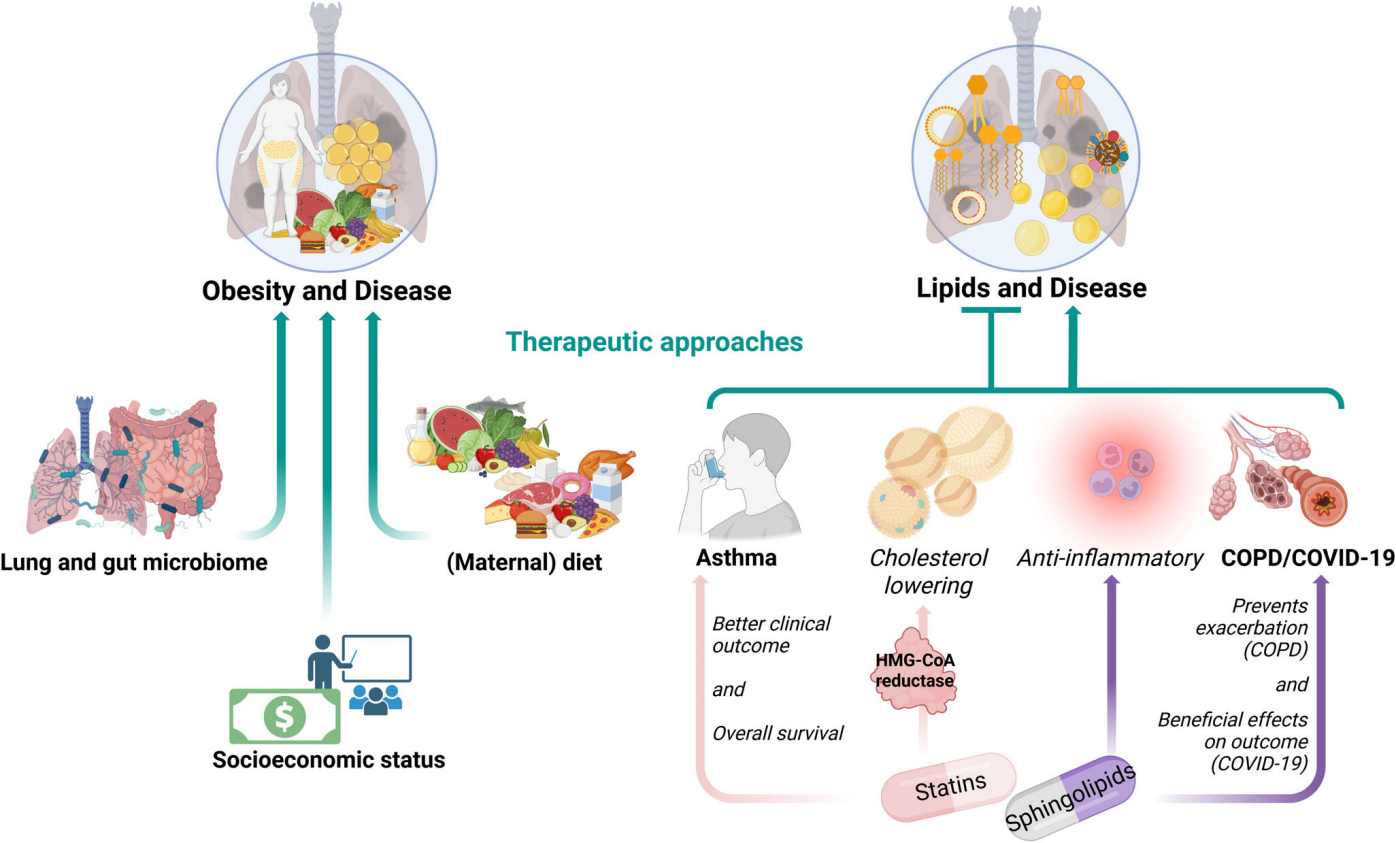
Overview of therapeutic strategies to target lipid metabolism in the context of lung pathologies. Created in BioRender. Alejandre Alcazar, M. (2026).

Over the last decades, numerous research studies focused on identifying possible therapeutic strategies to target lipids and their metabolism. Here, we highlight findings and new avenues that are already implemented or could be re‐purposed for lung diseases (Figure 4).

**Statins**, a class of cholesterol‐lowering drugs, reduce mortality from cardiovascular diseases and stroke. The beneficial effects of statins are primarily attributed to the inhibition of 3‐hydroxy‐3‐methylglutaryl coenzyme A (HMG‐CoA) reductase, a key enzyme in cholesterol biosynthesis, which causes a reduction of cholesterol levels in patients [216]. In lung disease, statins are controversially discussed. On the one hand, statin is associated with a lower risk of asthma exacerbation and a better clinical outcome in adult asthma [217]. There is also initial evidence that the treatment with statin at the time of IPF diagnosis is associated with improved overall survival [218]. On the other hand, statin‐induced lung disease (SILD) is a possible newly recognized side effect of statin therapy, a rare, but serious complication. However, the mechanism of lung injury underlying SILD is not yet defined [219].

**Sphingolipids**, which play a crucial role in various diseases, are also potential drug targets for treatment of pulmonary infections [220]. Sphingolipids with proinflammatory (e.g., ceramides) or anti‐inflammatory (e.g., S1P) functions compete with each other, and their relative abundance forms the basis of the so‐called, “sphingolipid rheostat”. The goal of various therapeutic strategies is to reduce ceramides or elevate S1P to shift the balance to an anti‐inflammatory environment. Possible ways to achieve this include sphingomyelinase inhibitors or N‐acetylcysteine to reduce ceramides, and supplementation with exogenous sphingosine or the S1P receptor agonist FTY7210 to increase S1P levels [221]. Based on this strategy, injection of S1P showed to be protective against mycobacterial growth in a mouse model of bacterial infection with *M. tuberculosis*. This effect was mediated by macrophage phospholipase D (PLD), favoring the acidification of mycobacteria‐containing phagosomes [222].

In COPD, elevated ceramides are a central mediator in disease manifestation. Thus, targeting the sphingomyelinase to reduce ceramide levels by *Functional Inhibitor of Acid SphingeMyelinAse* (FIASMAs) [223] or N‐acetylcysteine might prevent acute exacerbations of COPD [224]. In mice, treatment with the S1P precursor sphingosine, S1P agonist FTY720, or S1P receptor‐1 agonist SEW2871 resulted in S1P signaling recovery and protected against emphysema formation [225].

In addition, drugs and treatments targeting S1P and lipid metabolism have also gained attention in the context of viral infections like COVID‐19 [226]. In a clinical trial with acutely ill patients infected with SARS‐CoV‐2 and the need for respiratory support, re‐purposing the sphingosine‐1‐phosphate receptor ligand drug Ozanimod (FDA‐approved multiple sclerosis drug) showed that this new pharmacologic agent may safely be administered to patients hospitalized for viral pneumonianon, but nonsignificant association toward beneficial effects on disease outcome was observed [227]. Similarly, clinical trials investigating S1P‐based drugs including Fingolimod (FTY720, FDA‐approved multiple sclerosis drug) [228] and Opaganib (FDA‐approved cancer treatment) [229] were repurposed in COVID‐19 treatment with promising results.

The vast majority of research and therapeutic approaches target adults, but the lipid metabolism in infants and children and its implication in pulmonary diseases is less well studied. Since lung development is not completed at birth, the lungs of infants and children are highly susceptible and vulnerable to changes in lipid composition with subsequent aberrant lung morphogenic programming. Targeting lipid composition through dietary intervention already in utero may offer novel strategies to maintain physiological lung development and maturation for long‐lasting lung health across life span.

### 5.2 Maternal Diet, the Microbiome, and Immune Programming

The maternal diet has a significant impact on a child's health and disease risk. A gestational environment that promotes obesity directly compromises long‐term disease susceptibility, impairing the overall and respiratory health of offspring. For instance, maternal diets high in saturated fats are linked to an increased predisposition to lower respiratory tract infections in children [230]. Furthermore, maternal obesity and excessive gestational weight gain are associated with a higher risk of asthma diagnosis within the first seven years of life, as demonstrated in a large Danish cohort [231]. Conversely, a high‐quality maternal diet is intimately linked to a reduced risk of both underweight and obesity in offspring [232]. A diet that is high in unsaturated fats and dietary fiber, and low in saturated fats, sodium, refined grains and added sugars, appears to promote healthy weight outcome in childhood. [232]. Notably, stricter maternal sodium intake is strongly correlated with a lower risk of childhood overweight and obesity, likely due to sodium's role in promoting adipocyte expansion and fat accumulation.

These nutritional influences are further modulated by socioeconomic factors. Obesity and malnutrition are more prevalent among low‐income households and show a strong association with economic capacity [233]. A Spanish school‐based study revealed that children from lower‐income households exhibited poorer anthropometric profiles, including higher BMI, Z‐scores, triceps skinfold thickness, and waist and arm circumferences [233]. The same study reported that children from financially struggling families had a body weight that was 4.8 kg higher and were 3.6 times more likely to be obese. Therefore, improving the affordability of healthy, nutritious foods represents a clear opportunity for primary prevention with the potential to significantly reduce the burden of diet‐related diseases in socioeconomically disadvantaged populations.

**FIGURE 5:**
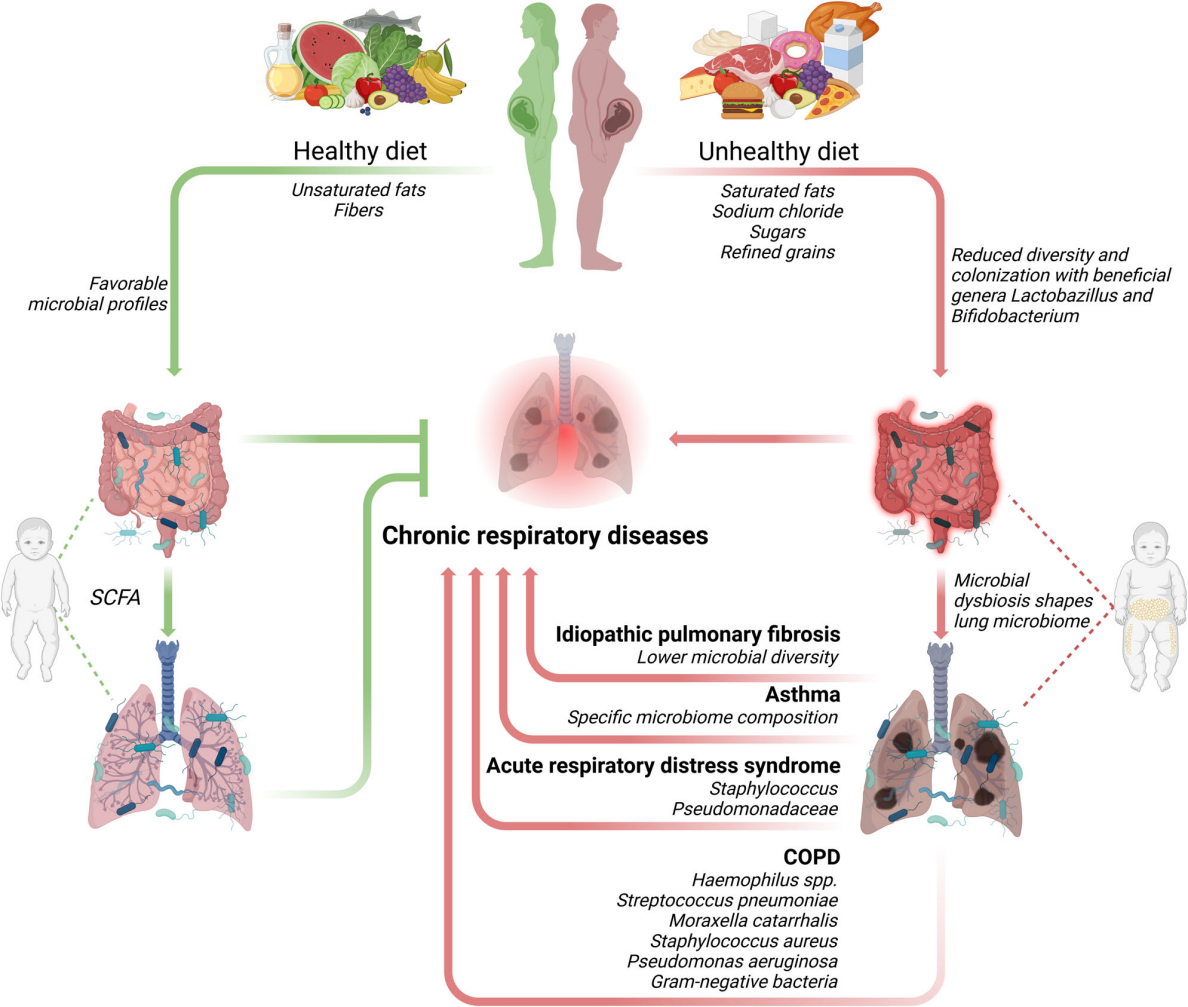
Overview of the interplay of gut and lung microbiome with lung homeostasis; SCFA, short‐chain fatty acid. Created in BioRender. Alejandre Alcazar, M. (2026).

Despite the mentioned direct effects of maternal nutrition, diet can profoundly affect microbial colonization and immune trajectory in the newborn (Figure 5). Although controlled feeding trials in pregnant women are rarely feasible, observational data consistently indicate that maternal diet, especially high‐fat diet, can shape the offspring's gut microbiome, a key player in immune system development [234, 235, 236]. The maternal microbiome serves as the primary microbial reservoir for the neonate, influencing initial colonization patterns and early immune priming. Accordingly, maternal nutritional habits directly impact the infant`s microbiome composition and ultimately immune function as well as disease susceptibility [234, 237, 238]. Notably, maternal consumption of a western‐style diet has been associated with reduced colonization of beneficial genera such as Lactobacillus and Bifidobacterium ‐ microbes linked to immune tolerance and reduced risk of IgE‐mediated disorders, including allergies [237, 239]. In contrast, maternal intake of fiber‐rich, plant‐based foods correlates with favorable microbial profiles in infants [240]. Microbial metabolites such as short‐chain fatty acids (SCFAs), particularly butyrate, act as critical immune regulators by balancing pro‐ and anti‐inflammatory responses and supporting mucosal immune integrity [241]. Conversely, maternal high‐fat diet depletes bacteroide species in neonates, and this dysbiosis persists beyond birth [239, 242]. Animal studies further demonstrate that sugar‐rich and unhealthy diets in early life reduce microbial diversity and promote the overgrowth of pathogenic bacteria [243].

Reduced microbiome diversity in infancy is increasingly recognized as a risk factor for chronic respiratory diseases, including asthma and COPD [244]. Multiple studies suggest that altered microbial composition and delayed microbial maturation during infancy contribute to heightened asthma risk later in childhood [245, 246, 247]. A well‐balanced, diverse microbiota during early life may promote immune tolerance and serve as a protective factor against chronic inflammatory respiratory conditions [244].

Beyond the gut microbiome, the lung microbiome ‐ and its interaction with the gut ‐ plays a crucial role in the maintenance of lung health and shaping disease progression. Accordingly, a fiber‐rich diet not only alter the intestinal microbiota but also influences the lung microbiota, which results in raised circulating SCFA levels, protecting against allergic airway inflammation [248, 249]. For several lung diseases microbial dysbiosis has been described as a common feature where a reduction in microbial diversity has been associated with disease progression [250, 251]. Some relevant lung‐related diseases will now be described in more detail:

In COPD, colonization of the lower airways with potentially pathogenic microbes (PPMs) correlates with more severe diseases symptoms. Clinical studies of the lung microbiome of patients with stable COPD revealed a strong association between PPMs (such as *Haemophilus* spp., *Streptococcus pneumoniae*, *Moraxella catarrhalis*, *Staphylococcus aureus*, *Pseudomonas aeruginosa*, and gram‐negative enteric bacteria) and neutrophilic inflammation along with elevated inflammatory cytokines (IL‐8, TNF) in the lower airways [252, 253]. A recent study of the lower airway microbiome in early‐stage COPD has shown that enrichment with oral commensals, including *Streptococcus*, *Staphylococcus*, *Prevotella*, and *Gemella*, is linked to lung function impairment and reduced response to bronchodilator therapy [254]. For asthma, certain microbiome compositions in the lower airway can be related to distinct clinical features like bronchial responsiveness or symptom severity [255, 256]. In IPF, a lower microbial diversity correlates with higher levels of proinflammatory and fibrotic mediators such as IL‐1Ra, IL‐1β, CXCL8, MIP‐1α, G‐CSF, and EGF [257, 258]. Additional research links lung dysbiosis to altered immune pathways in peripheral blood mononuclear cells, further supporting a key role for microbe–host interactions in the pathogenesis of pulmonary fibrosis [259]. Finally, a clinical study showed, that in acute respiratory distress syndrome (ARDS), enrichment of the lower airways with Staphylococcus or Pseudomonadaceae has been associated with intensified airway inflammation and poorer clinical outcomes, including lower 30‐day survival and prolonged mechanical ventilation [260]. Further emphasizing the importance of the lung microbiome, a 2016 clinical study showed that the presence of gut‐associated bacteria in the lower airways correlates with elevated alveolar TNF levels, a key driver of inflammation in ARDS. [261]. These findings underscore the microbiome as an important mediator of nutritional programming and highlight maternal and early‐life diet as modifiable determinants of respiratory health [262, 263, 264, 265, 266, 267, 268].

## 6 Conclusion

In summary, the present review shows the enormous importance of lipids, especially in the context of an obesogenic condition, with regard to lung health over the lifespan. A particular focus lies on the role of lipids, their mediators and lipid derivatives on lung development and maturation. This comprehensive literature review shows not only the importance of lipids in the maintenance of lung homeostasis and cellular integrity, but also how changes in the balance of different lipids adversely affect cellular function, immune response, and ultimately the risk and progression of lung disease at different stages of life. We have also focused on lipid‐related targets such as dietary interventions or pharmacological modifiers of lipid metabolism that may offer previously unexplored preventive and therapeutic opportunities.

## Conflicts of Interest

The authors have no conflicts to interest.

## Data Availability

Data sharing not applicable to this article as no datasets were generated or analysed during the current study.
